# Cefazolin-Loaded Double-Shelled Hollow Mesoporous Silica Nanoparticles/Polycaprolactone Nanofiber Composites: A Delivery Vehicle for Regenerative Purposes

**DOI:** 10.34172/apb.2023.032

**Published:** 2022-04-05

**Authors:** Negar Karimi, Mohsen Khorashadizadeh, Mohammad Yahya Hanafi-Bojd, Esmat Alemzadeh

**Affiliations:** ^1^Department of Medical Biotechnology, School of Medicine, Birjand University of Medical Sciences, Birjand, Iran.; ^2^Cellular and Molecular Research Center, Department of Medical Biotechnology, School of Medicine, Birjand University of Medical Sciences, Birjand, Iran.; ^3^Cellular and Molecular Research Center, Department of Pharmaceutics and Pharmaceutical Nanotechnology, School of Pharmacy, Birjand University of Medical Sciences, Birjand, Iran.; ^4^Infectious Diseases Research Center, Department of Medical Biotechnology, School of Medicine, Birjand University of Medical Sciences, Birjand, Iran.

**Keywords:** Adipose-derived stem cells, Double-shelled hollow mesoporous silica nanopartic, Electrospun nanofibers, Keratinocyte differentiation, Sustained drug release, Tissue engineering

## Abstract

***Purpose:*** As important challenges in burn injuries, infections often lead to delayed and incomplete healing. Wound infections with antimicrobial-resistant bacteria are other challenges in the management of wounds. Hence, it can be critical to synthesize scaffolds that are highly potential for loading and delivering antibiotics over long periods.

***Methods:*** Double-shelled hollow mesoporous silica nanoparticles (DSH-MSNs) were synthesized and loaded with cefazolin. Cefazolin-loaded DSH-MSNs (Cef*DSH-MSNs) were incorporated into polycaprolactone (PCL) to prepare a nanofiber-mediated drug release system. Their biological properties were assessed through antibacterial activity, cell viability, and qRT-PCR. The morphology and physicochemical properties of the nanoparticles and nanofibers were also characterized.

***Results:*** The double-shelled hollow structure of DSH-MSNs demonstrated a high loading capacity of cefazolin (51%). According to *in vitro* findings, the Cef*DSH-MSNs embedded in polycaprolactone nanofibers (Cef*DSH-MSNs/PCL) provided a slow release for cefazolin. The release of cefazolin from Cef*DSH-MSNs/PCL nanofibers inhibited the growth of *Staphylococcus aureus*. The high viability rate of human adipose-derived stem cells (hADSCs) in contact with PCL and DSH-MSNs/PCL was indicative of the biocompatibility of nanofibers. Moreover, gene expression results confirmed changes in keratinocyte-related differentiation genes in hADSCs cultured on the DSH-MSNs/PCL nanofibers with the up-regulation of involucrin.

***Conclusion:*** The high drug-loading capacity of DSH-MSNs presents these nanoparticles as suitable vehicles for drug delivery. In addition, the use of Cef*DSH-MSNs/PCL can be an effective strategy for regenerative purposes.

## Introduction

 Today, chronic wounds are considered an emerging epidemic and have created serious clinical challenges for patients. A significant problem with chronic wounds is microbial biofilm formation, which delays wound healing.^1-3^ Although the use of antibiotics has significantly reduced the risk of infections in patients, the widespread use of these drugs has increased the number of drug-resistant bacteria.^4^ Hence, it would be a top priority to discover novel methods for inhibiting bacteria.^1^

 Nanotechnology can help develop therapeutic strategies by designing drug delivery systems.^5-7^ Advantages of drug delivery systems include improved hydrophobic drug solubility, increased drug half-life, prolonged systemic turnover, a slow release, reduced drug dosage, and targeted delivery of drug compounds. These characteristics overcome the limitations of traditional therapeutic approaches.^8,9^ Among various delivery vehicles, mesoporous silica nanoparticles (MSNs) have gained much popularity in recent decades. Features such as high biocompatibility, high surface area, optimized mesoporous structure, and the ability to target and control drug release have proposed MSNs as promising materials for drug delivery.^10,11^ Electrospinning is another promising technique that plays a vital role in drug delivery.^12,13^ High encapsulation capacity, tunable porosity, and cost-effectiveness are some of the advantages of electrospun nanofibers in drug delivery.^1,14-17^

 As an attempt to assess the advantages of these two classes of nanomaterials, this study developed a double-shelled hollow MSNs-embedded nanofiber (DSH-MSNs/PCL) system to achieve a sustained antibiotic release. For this purpose, the study explored the cefazolin (Cef) release profile and antibacterial activity of nanofibers against *Staphylococcus aureus*. The potential of nanofiber mats for human adipose-derived stem cells (hADSCs) differentiation into keratinocyte cells was also evaluated after 21 days.

## Materials and Methods

###  Materials

 Cetyltrimethylammonium bromide (CTAB), polycaprolactone (PCL), tetraethyl orthosilicate (TEOS), 3-(4,5-dimethyl-2-thiazolyl)-2,5-diphenyl tetrazolium bromide (MTT), hydrochloric acid, 3-aminopropyltriethoxysilane (APTES), and acetic acid were provided from Sigma-Aldrich.^5^ Ammonia solution (32%), sodium carbonate (Na_2_CO_3_), ethanol (96%), and methanol (99.9%) were purchased from Merck (Germany). cDNA synthesis and RNA extraction kits were obtained from Parstous Co. (Mashhad, Iran). SYBR Green Real-Time-PCR Master Mix was provided from Ampliqon (Denmark). Fetal bovine serum (FBS), trypsin, penicillin, and streptomycin (P/S) were purchased from GIBCO (Grand Island, NY, USA). Dimethyl sulfoxide (DMSO) was supplied from Amresco (Solon, OH, USA). Lastly, the DMEM medium was purchased from Bio-Idea (Tehran, Iran).

###  Synthesis of DSH-MSNs

 DSH-MSNs were synthesized via previously established methods.^18^ First, TEOS was hydrolyzed using the Stöber method to prepare monodisperse solid silica spheres. Briefly, a mixed solution containing 10 mL of water, 3.15 mL of ammonia solution (25%), and 74 mL of ethanol was prepared to which TEOS (6 mL) was added. After 1 h, the white suspension was centrifuged at 12 000 rpm for 2 minutes and thoroughly rinsed with ethanol three times. The precipitation was dried at 60°C for 6 h.

 Later, 300 mg of the sample was dispersed in 60 mL of water and sonicated for 30 minutes. Subsequently, CTAB (450 mg), water (90 mL), ethanol (90 mL), and ammonia solution (1.65 mL) were added to the suspension and stirred for 30 minutes. 35 μL APTES and 750 μL TEOS were added and stirred for 6 hours. Then, the sample was centrifuged and washed with water and ethanol. The sediment was dried at 60°C for 6 hours. Afterward, the sample was dispersed in water (60 mL), and 1.2 g of anhydrous Na_2_CO_3_ was added to the solution. The mixture was stirred at 50°C for 10 hours and subsequently centrifuged and washed. The sedimentation was dispersed in ethanol again. Lastly, 0.1 mL of HCl was added to the sample and stirred at 60°C for 5 hours to eliminate CTAB. DSH-MSNs were obtained after centrifuging, washing, and drying.

###  Drug loading

 For drug loading into DSH-MSNs, different amounts of cefazolin (6, 9, 12, and 15 mg) were suspended in 1 mL of DSH-MSNs (3 mg/mL) aqueous suspension and stirred for 18-24 hours at 25°C. The Cef-loaded DSH-MSNs (Cef*DSH-MSNs) were then centrifuged for 2 minutes at 12 000 rpm and rinsed with water to remove free drug molecules. The sediment was dried in a desiccator.^19^ The loading content of Cef was measured using the standard curve of the Cef solution. The following formulae were used to determine the drug loading content and loading efficiency^19-21^:

 Drug loading content = (weight of the loaded drug in DSH-MSNs/weight of drug and DSH-MSNs) × 100% (Eq. 1)

 Drug loading efficiency = (weight of the loaded drug in DSH-MSNs/weight of feeding drug) × 100% (Eq. 2)

 The amount of Cef in the supernatant was measured by spectrophotometry at 270 nm.

###  Synthesis of nanofibers

 PCL solution (20%, viscosity: 27.8 cP) was prepared by dissolving PCL in acetic acid (90%) and stirring for 18-24 hours at 25°C. Subsequently, nanoparticles (DSH-MSNs and Cef*DSH-MSNs) and Cef were added to the PCL solution separately. In the next step, the prepared solutions were poured into a syringe (5 mL) with a needle tip (18G), and the fibers were collected on a grounded collector covered with aluminum foil. The optimal electrospinning parameters included a voltage of 14 kV, a 15 cm needle to collector distance, a flow rate of 1 mL/h, and a rotational speed of 200 rpm.

###  Physicochemical characterization

 The structure of Cef*DSH-MSNs, DSH-MSNs, Cef, Cef*DSH-MSNs/PCL, Cef*PCL, and PCL were evaluated with an FT-IR spectrometer (IR prestige-21, Shimadzu Co., Japan) in the spectral range of 400-4000 cm^-1^ using a potassium bromide disk (resolution of 4 cm^-1^). Next, 5-6 mg of samples were mixed, triturated with 100 mg potassium bromide, and placed in a sample holder for the potassium bromide disk. The surface morphologies of the DSH-MSNs and nanofibers were observed by SEM (FEI Quanta 450 Field Emission Scanning Electron Microscope, USA). The particle size of DSH-MSNs was characterized by a Transmission Electron Microscope Test Instrument (Carl Zeiss-EM10C-100 kV, Germany).

 The particle size distribution of DSH-MSNs dispersed in water was determined by DLS (Brookhaven, USA). A Zeta sizer Nano apparatus (Brookhaven, USA) was applied to measure the surface charge of the DSH-MSNs and Cef.

 The water contact angle (WCA) of nanofibers was measured by a WCA analyzer (Veho discovery VMS-004 Deluxe, England). Moreover, water droplets (1 μL) were pipetted onto the surface of PCL nanofibers before and after loading of DSH-MSNs.

###  Degradation study

 Hydrolytic degradation of the nanofibers was conducted in PBS solution (pH _= _7.4) for up to 4 weeks. The membranes were taken out from the PBS and characterized with SEM at week 4.

###  Mechanical properties and tensile strength of nanofibers

 Electrospun nanofiber mats’ mechanical properties and tensile strength play an essential role in tissue engineering. The mechanical properties of PCL nanofibers were evaluated before and after loading DSH-MSNs using a mechanical tensile testing device (Santam, STM-1, Iran). All nanofibers were cut in dimensions of 15 mm × 40 mm. The length of the nanofibers in the machine was 25 mm, and the tensile speed was 10 mm/min.

###  The cefazolin release profile of nanofiber mats

 As a measure to determine the cefazolin release profile, 20 mg (three replicates) of the electrospun nanofiber mats (Cef*DSH-MSNs/PCL and Cef*PCL) were weighted and sterilized with UV light for 20 minutes. Twenty mg of nanofibers were immersed in 3 mL PBS at pH 7.4 in a bain-marie at 37°C.^22^ The released medium was replaced with a fresh medium (3 mL) after the defined time intervals. Eventually, the concentrations of Cef were measured by UV/V spectroscopy at 270 nm.

###  Antibacterial assessment

####  Standard broth dilution method

 As a model for antibiotic drug, *S. aureus *(ATCC® 16538^TM^) was used to evaluate the antibacterial effects of Cef*DSH-MSNs/PCL, Cef*PCL, PCL, and DSH-MSNs/PCL. For quantitative analysis, 20 mg of the nanofiber mats were incubated in 5 mL of Luria broth (LB) medium containing *S. aureus* (OD: 0.3) at 37°C for 18-24 hours. Subsequently, 100 μL solution of each tube was diluted and cultured on an agar medium. The number of colonies was counted after incubating plates at 37°C for 17 hours. The bacterial suspension constituted the control group. All experiments were conducted in triplicate.

###  Disc diffusion method

 Disc diffusion test was performed to determine the growth-suppressing action of Cef*DSH-MSNs/PCL, Cef*PCL, PCL, and DSH-MSNs/PCL. In this method, 0.5 McFarland sterile normal saline suspension was prepared from freshly growing *S. aureus* (ATCC® 16538^TM^) culture. The punched electrospun nanofiber discs (6 mm) were placed on LB agar medium with the bacteria suspension (0.5 McFarland standard) and incubated at 37°C. A ruler was utilized to measure the inhibition zone in millimeters (mm). Three replicates were conducted under similar conditions for each sample.

###  Cell culture

####  Cell viability assay

 The toxicity of PCL and DSH-MSNs/PCL was determined using MTT assay on hADSCs. Human adipose-derived stem cells were kindly gifted by Dr. Mohsen Khorashadizadeh from Birjand University of Medical Science.^23^ For the MTT assay, 3 × 10^4^ cells/well were seeded in 24-well plates, treated with sterile nanofiber mats after 24 hours and incubated at 37°C with 5% CO_2_ for 72 hours. The medium was then substituted with 100 μL of MTT solution (1 mL fresh medium) and incubated at 37°C for 4 hours. For the formazan crystals formed by live cells to dissolve, the medium was removed entirely, and 500 μL of DMSO was added to each well plate. After 5 minutes, the absorbance was read on an ELISA plate reader at 570 nm.

 The cell viability (%) was calculated as follow:

 Viable cells (%) = abs_sample_ – abs_blank_/abs_control_ – abs_blank _× 100 (Eq. 3).

###  Cell seeding and differentiation of hADSCs into keratinocytes

 PCL and DSH-MSNs/PCL nanofibers were cut (circular disks in 15 mm diameter) and placed in a laminar flow hood to sterilize using UV radiation so as to prepare nanofiber mats for cell seeding. Afterward, the nanofiber mats were washed to remove any residual solvent. The nanofibers were immersed in DMEM overnight to facilitate cell attachment on the nanofiber surfaces. Next, 200 μL of cell suspension (cell density: 2 × 10^4^ cells/mL) were poured onto the surface of scaffolds and placed in an incubator for 4 hours (5% CO_2_ at 37°C). After the cells were attached to the nanofiber mats, the additional medium was added, and incubation was resumed. The fresh medium was replaced every two days during the incubation period, and this process was continued for 21 days.

###  Quantitative analysis of gene expression

 After 21 days, qRT-PCR was performed to assess the expressions of specific genes. Total RNA was extracted from cells using the Total RNA Extraction Kit (Parstous, Mashhad, Iran) as per the manufacturer’s protocol. Complementary DNA was prepared using the RevertAid First-Strand cDNA Synthesis Kit (Parstous, Mashhad, Iran) following the manufacturer’s instructions. For qRT-PCR, the amplification of the involucrin (IVL) and cytokeratin 18 (CK18) was performed using SYBR Green qRT-PCR Master Mix (A325402, Ampliqon, Denmark). Thermal cycling was carried out for 35 cycles of 30 seconds at 95°C, 1 minute at 60°C for IVL and 58°C for CK18, and 30 seconds at 72°C. Primer sequences were as follows: IVL, forward: 5′ CAGCACTCCACCAAAGCCTC 3′ and reverse: 3′ GCTCCTGATGGGTATTGACTG 5′; CK18, forward: 5′ TCGCAAATACTGTGGACAATGC 3′ and reverse: 3′ GCAGTCGTGTGATATTGGTGT; and GAPDH, forward: 5′ TGGACTCCACGACGTACTCAG 3′ and reverse: 3′ CGGGAAGCTTGTCATCAATGGAA 5′. The qRT-PCR data were analyzed by the 2^-ΔΔct^ method and normalized to glyceraldehyde-3-phosphate dehydrogenase.

###  Statistical analysis

 All data were summarized as mean ± SD, and statistical analysis was performed by *t* test (two-tailed) using GraphPad Prism (version 8.3.0) and REST (2009). A *P* value of less than 0.05 was considered to be statistically significant.

## Results and Discussion

###  Characterization of DSH-MSNs 

**Figure 1:**
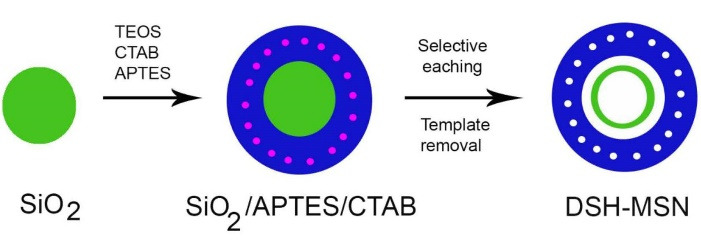
Schematic illustration of the preparation of DSH-MSNs.

**Figure 2:**
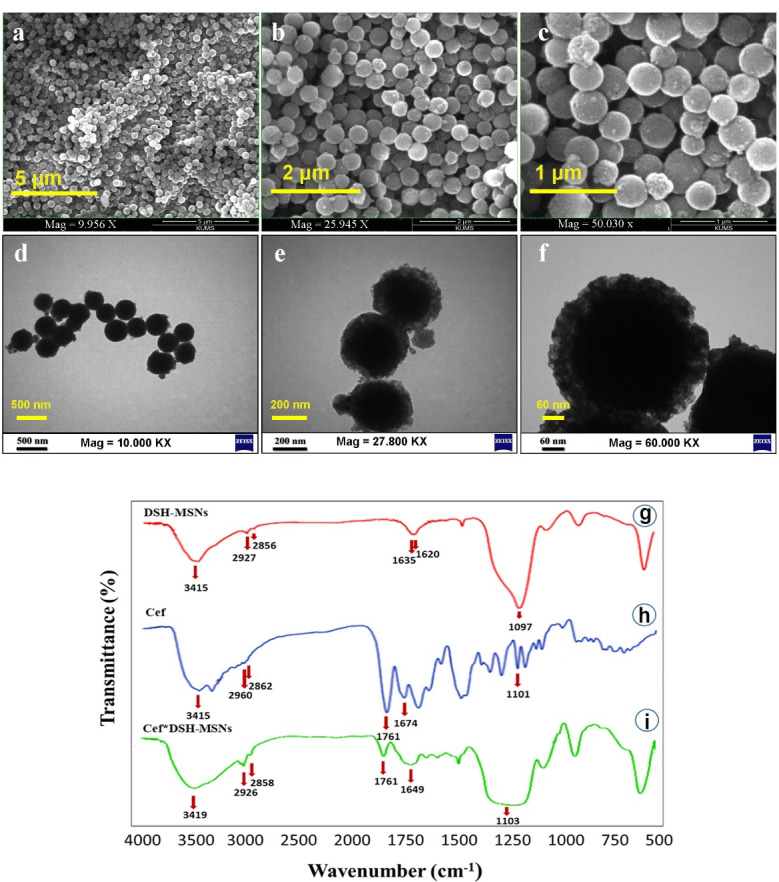
SEM (**a, b, c**) (HV: 30.00 KV, spot: 3.5, WD: 10.3 mm) and TEM (**d, e, f**) images of DSH-MSNs. Scale bars: (**a**) 5.0 μm; (**b**) 2.0 μm; (**c**) 1.0 μm; (**d**) 500 nm; (**e**) 200 nm; (**f**) 60 nm. FT-IR spectra of DSH-MSNs, Cef, and Cef*DSH-MSNs (**g-i**).

 DSH-MSNs were successfully synthesized by the Stöber method (Figure 1). DSH-MSNs had a round shape, an average particle size of approximately 400 nm, and a shell thickness of ~10 nm, as shown in SEM and TEM images (Figure 2a-f). The TEM image depicted a double-shelled structure, highly uniform size, and the shell thickness of nanoparticles (Figure 2f). The nanoparticles’ zeta potential (pH = 7.4) showed that DSH-MSNs were charged negatively (-17.31). The negative zeta potential of nanoparticles also revealed that the CTAB was removed from the structure.

 In this study, the adsorption method was used for loading drugs into the pores of MSNs. Accordingly, DSH-MSNs were immersed in the concentrated cefazolin solution. After 24 hours, the cefazolin molecules were absorbed on the pore walls. Drug-carrier interactions such as covalent bonding, electrostatic binding, hydrogen bonding, and van der Waals interactions facilitate the absorption of drug molecules in MSNs.^24,25^ Moreover, Cef loading into DSH-MSNs was achieved by hydrogen bonding interactions between the OH of DSH-MSNs (silica) and Cef. The FT-IR spectrum of DSH-MSNs (Figure 2g) showed sharp bands at 3415, 2927, 2856, 1635, 1097, 962, 800, and 472 cm^-1^.^18^ The FT-IR spectrum of Cef (Figure 2h) displayed sharp bands at 3415, 3284, 3143, 3059, 2960, 2862, 1761, 1674, 1600, 1550, 1489, 1282, 1240,1184, 1101, and 1064 cm^-1^.^26^ The sharp peaks at 3415, 3284, and 1600 cm^-1^ were related to the vibration of N-H stretching and the bending vibration of Cef.

 The FT-IR spectrum of the Cef*DSH-MSNs (Figure 2i) indicated sharp bands at 3419, 3304, 3005, 2926, 2858, 1761, 1649, 1625, 1544, 1490, 1103, and 958 cm^-1^ and broadband at 3600-2500 cm^-1^. The peaks at 3419, 3304, and 1597 cm^-1^ were related to N-H stretching and the bending vibration of Cef and DSH-MSNs’ second amine at the Cef*DSH-MSNs, respectively. Besides, the peaks at 1103 and 958 cm^-1^ were connected with the C-O stretching vibration of the Cef and DSH-MSNs, respectively. The FT-IR spectrum of the prepared Cef*DSH-MSNs indicated that the peak at 3005 cm^-1^ was characteristic of C-H stretching vibration, and the peaks at 1625, 1544, and 1490 cm^-1^ were related to C = C vinyl and aromatic stretching vibration of Cef. The peaks at 1761 and 1647 cm^-1^ were also correlated with C = O from Cef. The changes in the peak positions were observed from 3415 to 3419, 1674 to 1649, and 1600 to 1544 cm^-1^. These results confirm the presence of Cef and DSH-MSNs in the spectrum of Cef*DSH-MSNs. In addition, the results confirm the intermolecular hydrogen-bonding reaction between the OH of DSH-MSNs and Cef.

**Figure 3:**
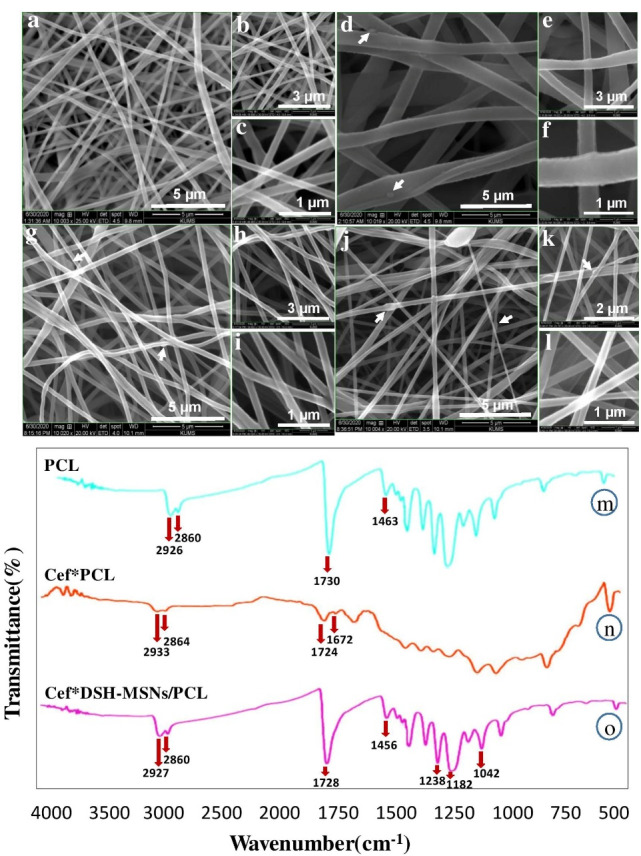
SEM images of PCL nanofibers. PCL (**a, b, c**), Cef*PCL (**d, e, f**), DSH-MSNs/PCL (**g, h, i**), and Cef*DSH-MSNs/PCL (**j, k, l**). Scale bars: (**a, d, g, j**) 5.0 μm; (**b, e, h**) 3.0 μm; (**k**) 2.0 μm; (**c, f, i, l**) 1.0 μm. FT-IR spectra of Cef*DSH-MSNs/PCL, Cef*PCL, and PCL (**m-o**).

 In this study, the Cef*DSH-MSNs were mixed with the PCL solution to synthesize the nanofibers. It should be noted that the acid pH of the solution has no negative impact on the stability of silica nanoparticles.^27^ In this regard, Hadipour Moghaddam et al demonstrated that the degradation rate of MSNs increased in alkaline conditions.^28^ Similarly, Ahmed and Day evaluated the stability of cefazolin in different pH levels, indicating a higher degradation of cefazolin in the alkaline pH.^29^ The FT-IR spectrum of PCL nanofibers was employed to investigate PCL, Cef*PCL, and Cef*DSH-MSN/PCL structures and the intermolecular hydrogen-bonding reaction between PCL and Cef. The FT-IR spectrum of Cef*DSH-MSNs/PCL (Figure 3o) indicated sharp bands at 3342, 3040, 2927, 2860, 1728, 1670, 1587, 1456, 1292, 1238, 1182, 1045, 960, and 731 cm^-1^. The broad peak at 2500-3500 cm^-1^ region also corresponded to OH stretching vibration group of carboxylic acid from Cef. The sharp peaks at 3342 and 1587 cm^-1^ were characteristic of N-H stretching vibration and the bending vibration of Cef at the Cef*DSH-MSNs/PCL.

The presence of the peak at 1728 cm^-1^ was correlated with C = O from Cef and PCL, and the peaks at 3342 and 1587 cm^-1^ were a feature of N-H stretching vibration and bending vibration of Cef, which can confirm the synthesis of Cef*DSH-MSNs/PCL nanofiber mats. Also, the FT-IR spectrum of Cef*DSH-MSNs/PCL showed prominent shifting of the peaks compared with those of PCL and Cef. The peaks at 1105, 880, 451 cm^-1^ were linked with Si–O–Si and Si–O stretching vibrations of DSH-MSNs, confirming the synthesis of Cef*DSH-MSNs. The peak positions shifted from 3400 to 3342, 1730 and 1724 to 1728, and 1591 to 1587 cm^-1^. These findings confirm the presence of PCL and Cef in the spectrum of Cef*DSH-MSNs/PCL and the synthesis of Cef*DSH-MSNs/PCL nanofiber mats.

**Figure 4:**
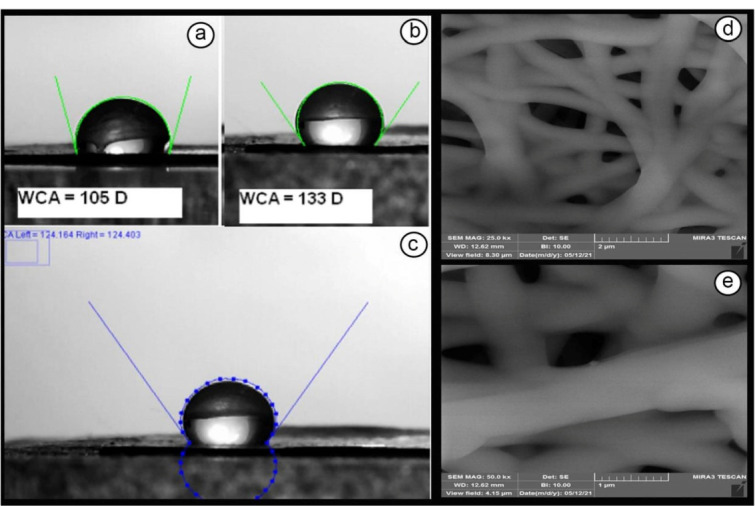
Water contact angle (**a**) Water drop on PCL nanofiber mats with 105 degrees (**b**) Water drop on DSH-MSNs/PCL nanofiber mats with 133 degrees (**c**) A water drop on the DSH-MSNs/PCL nanofiber mat surface showing contact angles of reflection below. (Note: water contact angle larger than 90 degrees, is considered hydrophobic). (**d, e**) FE-SEM images of *in vitro* degradation of electrospun PCL in PBS (pH_ = _7.4). Scale bars: (**d**) 2 μm; (**e**) 1 μm.

 WCA was calculated for PCL and DSH-MSNs/PCL nanofiber mats (Figure 4a-c). The hydrophobicity of pure PCL and DSH-MSNs/PCL nanofiber mats were 105 and 133 degrees, respectively. Figure 4c showed that the hydrophobicity of PCL nanofiber mats significantly increased after the addition of DSH-MSNs. Previous research also indicates that MSNs-embedded electrospun nanofibers can increase the hydrophobicity of nanofibers.^30^

 The degradability of PCL was evaluated after four weeks using FE-SEM (Figure 4d and 4e). After *in vitro* incubation in PBS, the diameter of PCL nanofibers increased, indicating that the swelling of nanofibers occurred during incubation.

###  Mechanical properties and tensile strength of nanofibers

 Electrospun nanofiber mats’ tensile strength and mechanical properties play an essential role in tissue engineering. The mechanical properties of PCL nanofibers were evaluated before and after loading DSH-MSNs using a mechanical tensile testing device (Santam, STM-1, Iran). All nanofibers were cut in dimensions of 15 mm × 40 mm. The length of the nanofibers in the machine was 25 mm, and the tensile speed was 10 mm/min.

**Figure 5:**
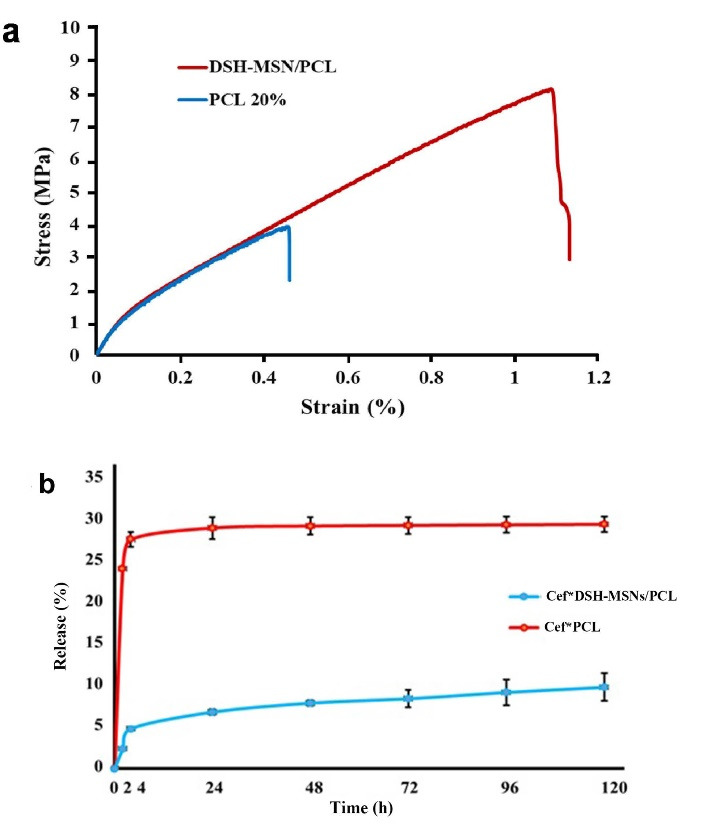
(**a**) Stress-strain curves of the PCL and DSH-MSNs/PCL nanofiber mats. (**b**) Release kinetics of cefazolin from Cef*DSH-MSNs/PCL and Cef*PCL nanofiber mats in phosphate buffer solution (PBS, pH = 7.4) (mean ± SD, n = 3).

Figure 5a and Table 1 present the typical stress-strain curves of the PCL and DSH-MSNs/PCL nanofiber mats. DSH-MSNs/PCL nanofibers have significantly higher mechanical properties than PCL ones. Indeed, when DSH-MSNs were added to PCL nanofibers, the nanofibers’ thickness and size uniformity increased, leading to higher adhesion forces (cohesion), which is ultimately associated with enhanced mechanical properties. Furthermore, the high affinity between DSH-MSNs and PCL matrix resulted in better dispersion of DSH-MSNs in nanofibers, which may explain such reinforcement stress transitions.^31^

**Table 1:** Tensile strength, young’s modulus, and elongation at break of the PCL and DSH-MSNs/PCL nanofiber mats

	**Young’s modulus (MPa)**	**Ultimate strength (MPa)**	**Elongation at break**
PCL	19.35 ± 2.74	3.89 ± 0.28	1.09 ± 0.12
DSH-MSNs/PCL	20.26 ± 1.69	8.02 ± 0.41	0.45 ± 0.7

 Gounani et al and Ganesh et al have demonstrated the increase in young’s modulus of PCL mats after silica nanoparticles are added.^32,33^ Similar to the case with PCL nanofibers, the MSNs embedded into PLGA nanofibers revealed an increase in young’s modulus and tensile strength.^34^ The similarity of the mechanical properties of scaffolds to those of the host tissue is one of the crucial goals of tissue engineering. Based on the values reported for tensile properties of different body tissues, the PCL and DSH-MSNs/PCL mats can serve as scaffolds in skin tissue engineering.^35^

###  The drug loading properties of Cef *DSH-MSNs/PCL and Cef *PCL

 As Table 2 indicates, the loading content and loading efficiency of DSH-MSNs were 10.27–51.79% and 5.72–26.85%, respectively. DSH-MSNs and Cef were used in the ratio of 3:12 to synthesize nanofiber mats. Some attractive properties such as large surface area and high pore volume introduce MSNs as excellent materials for loading drugs. Furthermore, a perfect mesoporous structure and the tunable pore size of MSNs help better control drug loading and release kinetics.^36^ Although MSNs have been broadly evaluated as drug delivery vehicles, the low drug-loading capacity remains the biggest challenge for these materials.^37^ As a strategy to solve this problem, hollow MSNs with a large cavity inside each original MSN have been developed to enhance the loading capacity of drugs.^37^ The high loading capacity of DSH-MSNs (51%) in this study indicates the practical application of DSH-MSNs as a drug delivery vehicle.

**Table 2:** Cef-loading content and loading efficiency of different concentrations

**DSH-MSNs**	**Cef**	**Drug loading content (w/w %)**	**Drug loading efficiency (%)**
3 mg	6 mg	10.27	5.72
3 mg	9 mg	27.41	12.58
3 mg	12 mg	51.79	26.85
3 mg	15 mg	31.99	9.41

###  The drug release properties of Cef *DSH-MSNs/PCL and Cef *PCL nanofiber mats

 For the Cef release profile, the drug-loaded nanofibers were soaked in PBS (pH_ = _7.4). Figure 5b illustrates the findings of the drug release profiles of Cef*DSH-MSNs/PCL and Cef*PCL nanofiber mats. The drug release curve of the samples showed that the release of Cef encapsulated in DSH-MSNs was slower than the Cef incorporated in PCL nanofibers. The drug release rate increased slowly after a few hours in the Cef*DSH-MSNs/PCL nanofiber mats, while the drug in the Cef*PCL nanofibers had a burst release. Multi-shelled hollow MSNs with high penetrating mesostructured shells sustain the drug release to a certain degree.^38^ However, an initial burst release is typically observed in these drug delivery systems.^18^ The incorporation of Cef*DSH-MSNs into PCL nanofibers revealed a more controlled release behavior. Drug release from pore DSH-MSNs and subsequently from nanofibers provide compelling evidence for the significantly slow release of Cef from Cef*DSH-MSNs/PCL nanofibers.^39^ This mechanism can prevent the initial burst release of the drug and increase the local concentration of the drug over time.^40^ Similarly, antibacterial tests confirmed a slow and controlled release from Cef*DSH-MSNs/PCL nanofiber mats. These preliminary results demonstrate the feasibility of using DSH-MSNs/PCL nanofibers for wound healing applications.

###  Antibacterial activity of Cef *DSH-MSNs/PCL and Cef *PCL nanofiber mats in broth microdilution under standard in vitro conditions

**Figure 6:**
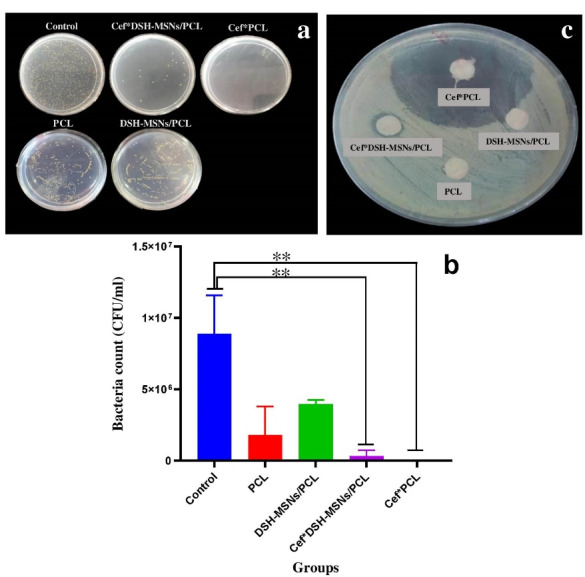
(**a, b**) Efficacy of Cef*DSH-MSNs/PCL, Cef*PCL against *S. aureus* (mean ± SD, n_ = _3). (Note: * indicates Cef*DSH-MSNs/PCL, Cef*PCL significantly different to the other groups. (* *P* < 0.05, ** *P* < 0.01). (**c**) *In vitro* antibacterial activity of Cef*DSH-MSNs/PCL, Cef*PCL against *S. aureus* (ATCC®16538^TM^). For PCL and DSH-MSNs/PCL there was no significant difference in the zone of inhibition.

 The antibacterial effects of PCL, DSH-MSNs/PCL, Cef*DSH-MSN/PCL, and Cef*PCL groups were determined via the standard broth dilution method. As Figure 6 depicts, Cef*DSH-MSN/PCL and Cef*PCL significantly decreased bacterial growth after 24 hours. Cef*PCL showed more lethality than Cef*DSH-MSNs/PCL, consistent with experimental data from the drug release profile. However, both groups demonstrated a significant difference from the control group (*P* < 0.05) (Figure 6a and 6b). The potential antibacterial activities of PCL, DSH-MSNs/PCL, Cef*DSH-MSNs/PCL, and Cef*PCL nanofiber mats were also evaluated against *S. aureus* (ATCC® 16538^TM^) through the disc diffusion method. As shown in Figure 6c, Cef*DSH-MSNs/PCL and Cef*PCL nanofiber mats inhibited the growth of *S. aureus* with inhibition zones of 9 mm for Cef*DSH-MSNs/PCL and 38 mm for Cef*PCL. For PCL and DSH-MSNs/PCL, there was no significant difference in inhibition zones. Infection, a significant problem in burn wounds, can effectively delay healing.^41^ Moreover, antibacterial resistance is one of the critical challenges for treating burn wounds. Although antibiotics significantly inhibit the growth of bacteria, high-dose antibiotic therapy may lead to toxic side effects in clinical practice.^42^ On the other hand, bacterial adhesion to the bandage surface and, eventually, biofilm formation are challenges that limit the applicability of some bandages.^43^

###  Cytotoxicity

**Figure 7:**
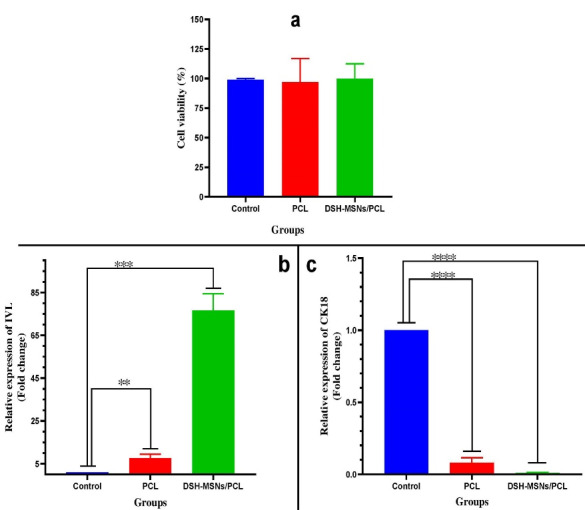
(**a**) Cell viability of hADSCs grown on PCL and DSH-MSNs/PCL (P < 0.05). Values are expressed as mean ± SD Modulation of IVL and CK18 profile by PCL and DSH-MSNs/PCL nanofiber mats: The mRNA levels of (**b**) Involucrin, (**c**) Cytokeratin18 at 21 days post-culturing, as determined by quantitative real-time PCR (Note: * indicates DSH-MSNs/PCL, PCL significantly different) (* *P* < 0.05, ** *P* < 0.01, *** *P* < 0.001, **** *P* < 0.0001).

 The hADSCs viability upon 72 hours exposure to DSH-MSNs and PCL nanofibers was quantified using MTT assay to evaluate possible cytotoxicity of the synthesized nanoparticles and nanofibers (Figure 7a). The nanoengineered membranes showed no toxicity to hADSCs, and cells were well grown on the surface of nanofibers without changes in morphology (*P* > 0.05). In agreement with our findings, numerous experiments investigating the cytotoxicity of silica nanoparticles in different cell lines have reported that silica nanoparticles have no toxic effects.^44-48^

###  Gene expression analysis

 To determine whether PCL and DSH-MSNs/PCL can stimulate the differentiation of hADSCs toward keratinocytes, we evaluated the expression of IVL and CK18 by qRT-PCR. As shown in Figure 7b, there was a significant increase in the mRNA expression level of IVL in the PCL and DSH-MSNs/PCL nanofiber mats compared with the control group after 21 days (*P* < 0.05). As against the PCL group, the IVL expression was significantly high in the DSH-MSNs/PCL group (*P*< 0.05). However, the qRT-PCR data in Figure 7c exhibited a significant reduction in the mRNA expression level of CK18 in the PCL and DSH-MSNs/PCL groups after 21 days (*P* > 0.05). In this regard, Yang et al also demonstrated that the expression of CK18 in the absence of an induction medium was negative.^44,49^

 Epithelialization is a vital stage in wound healing, and keratinocytes play a crucial role in promoting re-epithelialization.^50^ Poor quality and insufficient quantity of keratinocytes in unfavorable conditions such as severe burns may impair wound healing.^51^ Differentiating hASCs toward keratinocytes can be a good solution for promoting burn wound healing. Few researchers have demonstrated the capacity of hADSCs in epidermal differentiation using nanoparticles and scaffolds.^52,53^ According to previous studies, cells often need an induction medium for differentiation.^54^ In our study, the differentiation effect of synthesized 3D scaffolds on hADSCs into keratinocytes was assessed without using an induction medium.

 The results of qRT-PCR revealed that the IVL expression level in the presence of scaffolds (DSH*MSNs/PCL, PCL) was significantly increased. Notably, the DSH-MSNs/PCL demonstrated a substantially influential role in IVL modulation compared to PCL nanofiber mats. In this regard, several studies have shown that nanoparticles can easily insert into cell membranes, locate in the cytoplasm, affect cellular signaling pathways, and induce differentiation by virtue of their small size.^54^ Based on Wei and colleagues’ findings, silica nanoparticles’ unique biological and mechanical properties are another possible contributor to stem cell differentiation.^54^ Nanofiber scaffolds also facilitate the differentiation of ASCs into epidermal cells by mimicking the structure and function of ECM.^52^ Alongside this, Ganesh et al demonstrated, for the first time, that electrospun MSNs/PCL nanofiber mats could induce differentiation of hMSCs to osteogenic cells.^32^ It should be noted that three-dimensional culture provides the biochemical aspects of cell-cell communication, signaling mechanisms, plasticity, cell proliferation, and migration to induce differentiation.^55,56^ Besides, scaffolds provide conditions for cell growth and differentiation into keratinocytes by mimicking the extracellular matrix and creating a 3D environment.^57^

 The large cavity of DSH-MSNs not only increases the loading capacity but also prolongs the release profile of the drug. The differentiation effect of DSH-MSNs and PCL nanofibers on hADSCs into keratinocytes is another reason that considers DSH-MSNs as a good strategy for promoting wound healing. Our findings are promising and should be explored with other tests. Future work should focus on loading the two drugs, evaluating the long-term toxicity of DSH-MSNs, and assessing their impact on wound healing in animal models.

## Conclusion

 This study has developed a composite nanofibrous material for controlled and sustained drug release. The incorporation of Cef*DSH-MSNs into PCL nanofibers significantly reduced the burst release of the cefazolin and led to a sustained drug release. The drug-loaded nanofiber mats were effective against *S. aureus* and significantly inhibited their growth. The DSH-MSNs/PCL also significantly stimulated the differentiation of human adipose stem cells to keratinocytes. Hence, the Cef*DSH-MSNs/PCL nanofiber mats demonstrated their potential in drug delivery and regenerative medicine.

## Acknowledgments

 The office of Vice-Chancellor for Research and Technology, Birjand University of Medical Sciences, supported his research project (grant number: Ir.bums.456090).

## Competing Interests

 There is no conflict of interest to declare.

## Ethical Approval

 Ethical approval was granted by Ethics Committee of Birjand University of Medical Sciences (Ethics No. IR.BUMS.REC.1398.408).
